# Oral and Maxillofacial Pathology in Latin America and the Caribbean: A Comprehensive Survey of Recognition, Training, and Practice

**DOI:** 10.1111/jop.70051

**Published:** 2025-09-03

**Authors:** Leonor Victoria González‐Pérez, Cristina Saldivia‐Siracusa, Ana Carolina Prado Ribeiro e Silva, Lady Paola Aristizábal Arboleda, Karen Patricia Domínguez Gallagher, Anna Luiza Damaceno Araújo, Pablo Agustin Vargas, María Luisa Paparella, Ana Verónica Ortega Pinto, Wilfredo Alejandro González‐Arriagada, Claudia Patricia Peña‐Vega, Roberto Gerber‐Mora, Gilda Lucia García Heredia, Florence Juana Maria Cuadra Zelaya, Ileana del Rosario Hurtado Castillo, Arvind Babu Rajendra Santosh, Adalberto Mosqueda‐Taylor, Erick Antonio Castillo Gurdián, María del Carmen González Galván, Wilson Delgado Azañero, Helen Rivera, Ronell Bologna‐Molina, Mariana Villarroel‐Dorrego, Janeth Liliam Flores Ramos, Carlos Alberto Gaidos Nates, Patricia Reiván Ortiz, Claudette Arambu Turcios, Erick Martínez Cruz, Loyden Evan Ken, Keith D. Hunter, Alan Roger Santos‐Silva

**Affiliations:** ^1^ Departamento de Diagnóstico Oral, Faculdade de Odontologia de Piracicaba University of Campinas (UNICAMP) São Paulo Brazil; ^2^ Faculty of Dentistry University of Antioquia Medellín Colombia; ^3^ Dental Oncology Service Instituto do Câncer do Estado de São Paulo, ICESP‐FMUSP São Paulo Brazil; ^4^ Oral Medicine Service Sírio Libanês Hospital São Paulo Brazil; ^5^ Graduate Program of A. C. Camargo Cancer Center São Paulo Brazil; ^6^ Department of Oral Medicine and Oral Pathology, School of Dentistry National University of Asunción (UNA) Asunción Paraguay; ^7^ Head and Neck Surgery Department University of São Paulo Medical School São Paulo Brazil; ^8^ Surgical Pathology Laboratory, Oral Pathology Department, Faculty of Dentistry University of Buenos Aires Buenos Aires Argentina; ^9^ Department of Oral Pathology and Medicine, Faculty of Dentistry Universidad de Chile Santiago Chile; ^10^ School of Dentistry Universidad de Los Andes Santiago Chile; ^11^ Department of Oral and Maxillofacial Pathology El Carmen Hospital, MD Luis Valentín Ferrada Maipú Chile; ^12^ Dentistry Department National University of Colombia Bogotá Colombia; ^13^ Pathology Service University Hospital of the National University of Colombia (HUN) Bogotá Colombia; ^14^ Pathology Service Colsanitas Clinic—Keralty CEPAT Bogotá Colombia; ^15^ Sección de Diagnóstico, Facultad de Odontología Universidad de Costa Rica San José Costa Rica; ^16^ Head and Neck Service National Institute of Radiobiology and Oncology La Habana Cuba; ^17^ Department of Pathology, School of Dentistry University of El Salvador San Salvador El Salvador; ^18^ School of Dentistry University of San Carlos Guatemala City Guatemala; ^19^ Faculty of Medical Sciences, School of Dentistry The University of the West Indies Kingston Jamaica; ^20^ Health Care Department Metropolitan Autonomous University Mexico City Mexico; ^21^ Universidad Americana Managua Nicaragua; ^22^ Department of Oral Pathology, Oral Medicine and Oral Surgery, School of Dentistry Universidad Peruana Cayetano Heredia Lima Peru; ^23^ School of Dentistry Universidad Iberoamericana (UNIBE) Santo Domingo Dominican Republic; ^24^ Histopathology Laboratory Dermatological Institute Dr Huberto Bogaert Santo Domingo Dominican Republic; ^25^ Molecular Pathology Area, School of Dentistry University of the Republic Montevideo Uruguay; ^26^ Laboratory of Oral Histopathology Central University of Venezuela (UCV) Caracas Venezuela; ^27^ Dentistry Department Universidad Mayor de San Andrés (UMSA) La Paz Bolivia; ^28^ Dentistry Department Universidad del Valle (UNIVALLE) La Paz Bolivia; ^29^ Service Oral Pathology Simon Bolivar Hospital Bogotá Colombia; ^30^ Viña del Mar University Valparaíso Chile; ^31^ Universidad Católica de Honduras Tegucigalpa Honduras; ^32^ Ministry of Health Panama; ^33^ Belize National Pathology Centre Ltd. Belize Medical Doctors and Dentists Association (BMDA) Belmopan Belize; ^34^ Liverpool Head and Neck Centre, Molecular and Clinical Cancer Medicine University of Liverpool Liverpool UK

**Keywords:** Caribbean region, Latin America, oral and maxillofacial pathology, pathologists, professional practice, specialty boards, training programs

## Abstract

**Background:**

Oral and maxillofacial pathology (OMFP) is a dental specialty that studies the causes, processes, and effects of diseases in the oral and maxillofacial area, while also contributing to diagnosis and treatment. Its recognition, training, and professional practice vary across the globe. This study aimed to explore the training and professional development of OMFP in Latin America and the Caribbean (LAC), including important issues such as specialty recognition, service regulation, postgraduate education, number of specialists, career opportunities, and perceived barriers.

**Methods:**

An observational, cross‐sectional study was conducted, where senior professionals in OMFP from 21 LAC countries were invited to complete a self‐administered questionnaire via the REDCap web platform.

**Results:**

Experts from 21 countries reported recognition of OMFP as a dental specialty in 76.2% of the countries, with 61.9% offering it as an independent program distinct from oral medicine. Specific regulations for practice were present in 52.4% of the countries, and 33.3% offered postgraduate programs, mainly combining other specialties. The professional activities of participants were diverse, including roles in private practice, universities, research, laboratories, and hospitals. However, 61.9% of participants identified the lack of recognition for multidisciplinary teams as a significant barrier to their practice.

**Conclusion:**

This groundbreaking study provides an overview of key aspects of training, practice, and recognition of OMFP in 21 LAC countries. The findings demonstrate significant variation both within the region and when compared to global studies, providing a crucial foundation for future research in this area.

## 1 Introduction

Oral and maxillofacial pathology (OMFP) is a dental specialty and a branch of general pathology that focuses on the nature, classification, and treatment of oral and maxillofacial complex diseases, exploring their causes, processes, and consequences. It is essential for accurate diagnosis, early detection, and informed treatment planning, as well as for research, innovation, and integration between oral and general health, representing a vital role for improvement in quality of life [1, 2].

OMFP is at the intersection between basic and clinical sciences. Its practice includes histopathological and immunohistochemical diagnosis of biopsy samples, fine needle aspiration, and exfoliative cytology. It also correlates histomorphological features with clinical and radiological findings, along with guiding direct treatment (clinical oral pathology) or indirect guidance in patient management (anatomical oral pathology) [3, 4].

The importance of this field has been widely acknowledged after Pierre Fauchard's era and its official recognition as a dental specialty by the American Dental Association in 1950 [5]. Diverse postgraduate training programs have been developed globally [6, 7], and multinational and regional scientific organizations have been established [8]. However, significant challenges remain, including formal acknowledgment of the specialty, lack of standardized training frameworks and best practices, and proper quality management in laboratories [3]. These gaps adversely affect professional growth, patient care quality, and health systems efficiency [6, 7].

Latin America and the Caribbean (LAC) is a region characterized by cultural and ethnic diversity, emerging economies, increasing political instability, and significant inequality. The region's fragmented health systems pose substantial challenges for equitable and high‐quality services [9]. While health science education has expanded significantly in recent decades, the human resources' supply remains unregulated. In many countries, health authorities lack adequate information and methodologies to effectively evaluate human resources, hindering informed decision‐making [10]. Hence, there are limited studies assessing the status of OMFP in LAC.

This study explores the profile of OMFP professionals in LAC through their academic training and professional trajectories. It also examines postgraduate programs availability, quality assurance policies, specialty recognition, and barriers to its practice, aiming to produce regional evidence that founds and guides the specialty's regional and global development.

## 2 Materials and Methods

### 2.1 Study Design and Ethical Considerations

This cross‐sectional, observational study complies with the Declaration of Helsinki, and its protocol was approved by the Research Ethics Committee of the Piracicaba Dental School, University of Campinas (FOP‐UNICAMP) (approval number: 55115521.3.0000.5418). Informed consent was obtained from every participant.

### 2.2 Collection Instrument

The survey included two main sections: individual questions covering demographic aspects, and general questions about the specialty. To address the linguistic diversity of the region, the instrument was developed in both English and Spanish. Two independent bilingual researchers translated the text, and then an external translator conducted an additional review. Any discrepancies were resolved through discussion.

The self‐administered questionnaire was made available on the FOP‐UNICAMP REDCap platform (Version 13.8.1, Vanderbilt University, Nashville, Tennessee, USA), a validated, secure, and versatile tool for survey‐based research [11, 12]. Subsequently, some members of the multinational team assessed validity, comprehension, and feasibility through an internal pilot test [13]. A formal invitation was sent to each potential participant. They received the survey link (https://redcap.fop.unicamp.br/redcap/surveys/?s=LH4HWRXH4M8TWNMK) to agree, sign the informed consent form, and fill in the questionnaire. Those who did not respond got their e‐mail addresses verified and were sent up to two reminder e‐mails with a response period of 15 calendar days (from February 22 and October 10, 2022).

### 2.3 Sample Selection Criteria and Recruitment

A non‐probability purposive sampling approach was employed, using both direct and indirect approaches for recruitment [14] to target key senior OMFP professionals with substantial knowledge and expertise. These contacts were obtained through scientific networks, international academic organizations, and personal contacts of the researchers. The study included all 33 countries defined by the Pan American Health Organization (PAHO) as part of the LAC region (Supporting Information S1). Efforts were made to recruit at least one OMFP specialist per country. An OMFP senior expert was defined as a dentist prepared with OMFP‐focused postgraduate training (residencies, specialties, diplomas, master's and/or doctoral degrees). As an alternative for countries where participants with these requirements could not be found, pathologists with experience in interpreting oral and maxillofacial biopsies were included (Supporting Information S2). Exclusion criteria fit for those participants with no current professional practice in LAC and incomplete questionnaires. When there was more than one participant per country, the answers to the individual questions were retained, and the general questions of the specialty were merged.

### 2.4 Data Analysis

Two researchers (L.V.G.‐P. and C.S.‐S.) organized and analyzed data, addressing uncertainties through direct communication with respondents. When participants could not distinguish OMFP from oral medicine (OM) or provided ambiguous responses, these were treated affirmatively and subjected to comprehensive analysis. The collected data was compiled using Microsoft Excel version 2110 (Microsoft Office LTSC Professional Plus 2021, Microsoft Corp., Washington, USA). Descriptive and quantitative analyses were conducted to evaluate categorical and continuous variables, employing mean, median, range, and frequency percentage values. All statistical analyses were performed using SPSS version 25 (SPSS Inc., Chicago, USA).

## 3 Results

**FIGURE 1:**
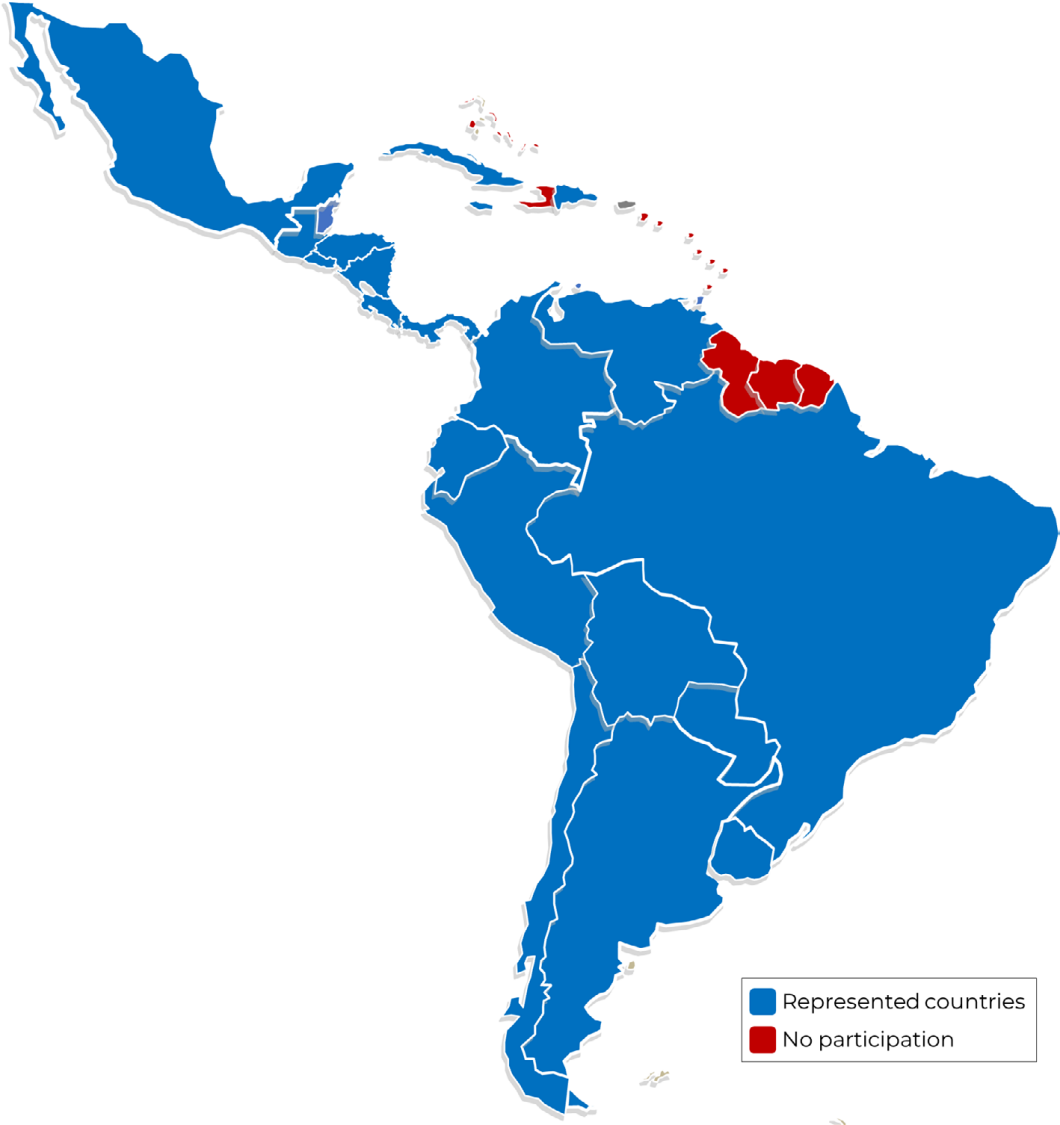
Distribution of participants by country.

During the study period (February–October 2022), potential participants were contacted in 21 of the 33 countries, and 23 surveys were fully completed. The countries represented are shown in Figure 1 and the detailed list of participants in Supporting Information S2. For countries with more than one participant (Chile and Colombia), the criteria for sample selection were applied.

### 3.1 Demographics and Professional Profile of Respondents

Supporting Information S3 provides detailed demographic and professional information about the 23 participants. Sex distribution had a proportion of 13 (56.5%) females and 10 (43.5%) males. The overall mean age was 48.8 years (range 31–76 years). All participants had varying levels of postgraduate training; particularly, 18 (78.3%) had specialization (of them, 47.8% in OMFP), 11 (47.8%) had a master's degree (26.1% in OMFP), and 8 (34.8%) had a PhD (26.1% in OMFP). In addition, 19 (82.6%) participants received academic training in international institutions, while 18 (78.3%) reported having received complementary interdisciplinary training.

Most participants (87%) are employed in their home countries, 22 (95.2%) work at histopathology laboratories (public and private), and 19 (82.6%) work in universities.

In 13 diagnostic centers (56.5%), less than 500 samples were processed annually, and each participant signed 50 or fewer histopathology reports per month. Additionally, payment systems for histopathology analyses varied, most commonly being a combination of private funding, consortia, and health insurance (Supporting Information S4).

### 3.2 Characterization of the Oral and Maxillofacial Pathology Specialty: Recognition, Quality Regulation, and Professional Associations

Governmental and nongovernmental agencies recognized OMFP as a dental specialty in 16 (76.2%) LAC countries. Brazil was the first country to grant official recognition in 1971, while Nicaragua was the most recent in 2020. OMFP is accepted as an independent professional field in 13 (61.9%) LAC countries. However, in Colombia, Chile, Guatemala, Honduras, Nicaragua, Paraguay, and Peru, the professional practice is integrated with other dental areas (Table 1) (Supporting Information S5).

**TABLE 1:** General characteristics of the OMFP specialty in LAC countries.

Characteristics	*N* (%)
Total	21 (100)
*Official recognition of the specialty*	
In your country, is the oral and maxillofacial pathology specialty officially recognized by any local registering authorities?	
Yes	16 (76.2)
No	5 (23.8)
Mean (recognition year)	1993
Range (years)	1971–2016
Who regulates/recognizes/authorizes the oral and maxillofacial pathology specialty in your country?^a^	
Ministry of Health	9 (42.9)
Ministry of Education	4 (19.0)
Faculties/Associations/Federations	12 (57.2)
Does not know/does not answer	2 (9.5)
Other	1 (4.7)
Is oral and maxillofacial pathology an independent field from oral medicine?	
Yes	13 (61.9)
No	7 (33.4)
Does not know/does not answer	1 (4.7)
*Quality standards of professional activities*	
Are there any standards/policies/regulations to perform oral and maxillofacial pathology?	
Yes	11 (52.4)
No	9 (42.9)
Does not know/does not answer	1 (4.7)
Who is responsible for quality assurance regarding the clinical practice of oral and maxillofacial pathology specialists in your country?	
Ministry of Health	6 (28.6)
Ministry of Education	0 (0.0)
Faculties/associations/federations	3 (14.3)
Does not know/does not answer	2 (9.5)
There's no specific control	10 (47.6)
Other	0 (0.0)
Do oral and maxillofacial pathology specialists must do a recertification exam?	
Yes	0 (0.0)
No	18 (85.7)
Does not know/does not answer	3 (14.3)
*Academic/professional/scientific groups*	
Are there any national associations/federations/societies in your country that group oral and maxillofacial pathology practitioners?	
Yes	10 (47.6)
No	11 (52.4)
Does not know/does not answer	0 (0.0)
Detailed information is provided in Supporting Information S5	
*Postgraduate training and definition of competencies*	
Are there oral and maxillofacial pathology postgraduate courses in your country?	
Yes	7 (33.3)
No	14 (66.7)
Does not know/does not answer	0 (0.0)
Is the oral and maxillofacial pathology postgraduate course a unique field of study or is it combined with other area(s)?^a^	
Single	4 (19.0)
Combined	5 (23.8)
Does not apply	12 (57.2)
What are the types of oral and maxillofacial pathology postgraduate courses available in your country?^a^	
Specialization	7 (33.4)
Master's degree	3 (14.3)
Doctoral degree	2 (9.5)
Residency	1 (4.7)
Others (courses/trainings/diplomas)	12 (57.2)
Type of training at oral and maxillofacial pathology and duration in months	
Specialization	24 (12–36)
Master's degree	24
PhD	36–48
Residencies	36
Others (courses/training/diplomas)	6 (1–12)
Are there any government funding resources for academic training on oral and maxillofacial pathology in your country?	
Yes	12 (57.2)
No	8 (38.1)
Does not know/does not answer	1 (4.7)
Are the competencies/capacities of the oral and maxillofacial pathologist defined?	
Yes	11 (52.4)
No	8 (38.1)
Does not know/does not answer	2 (9.5)
Who defines oral and maxillofacial pathology practitioners' competencies?^a^	
Government	5 (23.8)
Universities	6 (28.6)
Associations/federations/colleges	4 (19.0)
Other	0 (0.0)
Which areas are considered competencies of an oral and maxillofacial pathologist in your country?^a^	
Oral and maxillofacial complex	21 (100.0)
Ear, nose, and throat	2 (9.5)
Head and neck	4 (19.0)
Other (dermatology)	1 (4.7)
*Number of specialists and space for professional performance*	
How many certified oral and maxillofacial pathology specialists do you estimate that work in your country?	
0–5	12 (57.2)
6–10	3 (14.3)
11–20	2 (9.5)
21–50	2 (9.5)
> 100	2 (9.5)
Which are the possible work fields available for oral and maxillofacial pathology practitioners in your country?^a^	
Public universities	19 (90.5)
Private universities	16 (76.2)
Public hospitals	9 (42.9)
Private hospitals	9 (42.9)
Public practice/laboratory	7 (33.4)
Private practice/laboratory	14 (66.7)
Research at public institution	13 (61.9)
Research at private institution	11 (52.4)
Other	0 (0.0)

^a^ Participants were able to select more than one answer.

In 9 (42.9%) countries, OMFP practice is regulated to ensure the quality and efficacy of clinical and laboratory procedures. In 6 (28.6%) of these regulated countries, the Ministry of Health establishes the standards, while in 3 (14.3%), it is faculties, associations, or federations; albeit no certification exams are mandatory for professionals in the region (Table 1).

In this study, less than half of LAC countries (47.6%) had associations, federations, or societies representing OMFP. Interestingly, 80% of these entities were active on social media platforms, and there were 2 (20%) societies with scientific journals (Brazil and Nicaragua) (Supporting Information S5).

### 3.3 Academic Training Programs in Oral and Maxillofacial Pathology in the Region

In 11 (52.4%) of the countries, the competencies for OMFP were clearly defined. Only 7 (33.3%) countries offered some kind of postgraduate program in OMFP; Argentina and Bolivia had standalone training programs, while Brazil, Colombia, and Peru offered combined programs integrating other dental specialties such as OM, oral surgery, and radiology, and Chile and Mexico provided both types of programs (Table 1) (Supporting Information S6).

Specialization programs were the most common, lasting from 12 to 36 months, while PhD programs, offered only in Mexico and Brazil, were reported to last 36 and 48 months, respectively. Additionally, master's and doctoral studies were publicly funded in 57.2% of the countries (Table 1).

### 3.4 Number of Specialists and Professional Practice in LAC Countries

**FIGURE 2:**
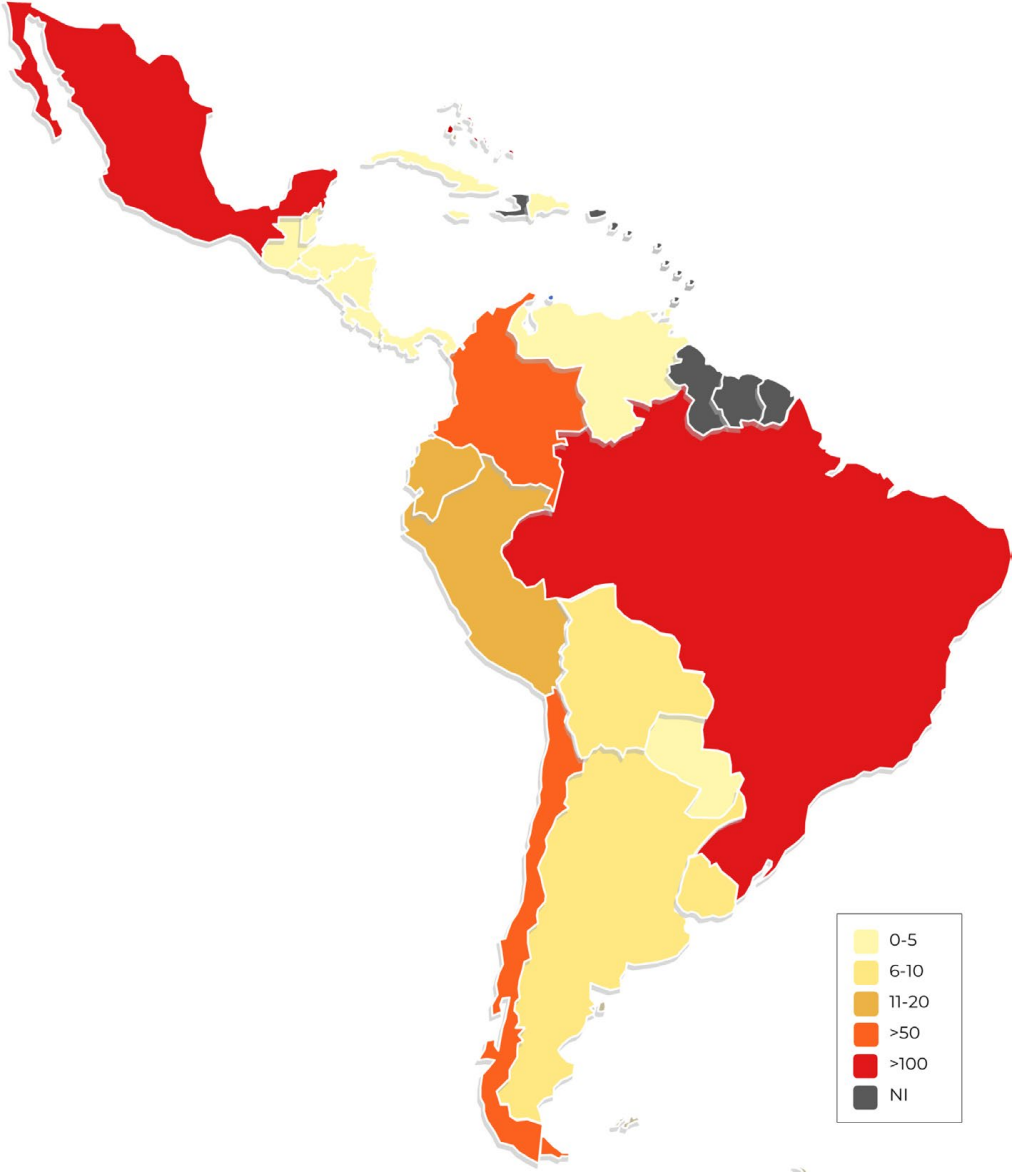
Heatmap depicting the number of OMFP professionals by participant country.

The distribution of specialists in OMFP differs significantly across LAC countries. There are fewer than 5 specialists in 12 (57.2%) countries and only Brazil and Mexico have more than 100 OMFP (Figure 2).

Additionally, it was found that OMFP practitioners identified universities, both public (90.5%) and private (76.2%), as the most common professional environments, followed by private practice (66.7%) and research at public institutions (61.9%) (Table 1).

### 3.5 Barriers to Professional Practice

**FIGURE 3:**
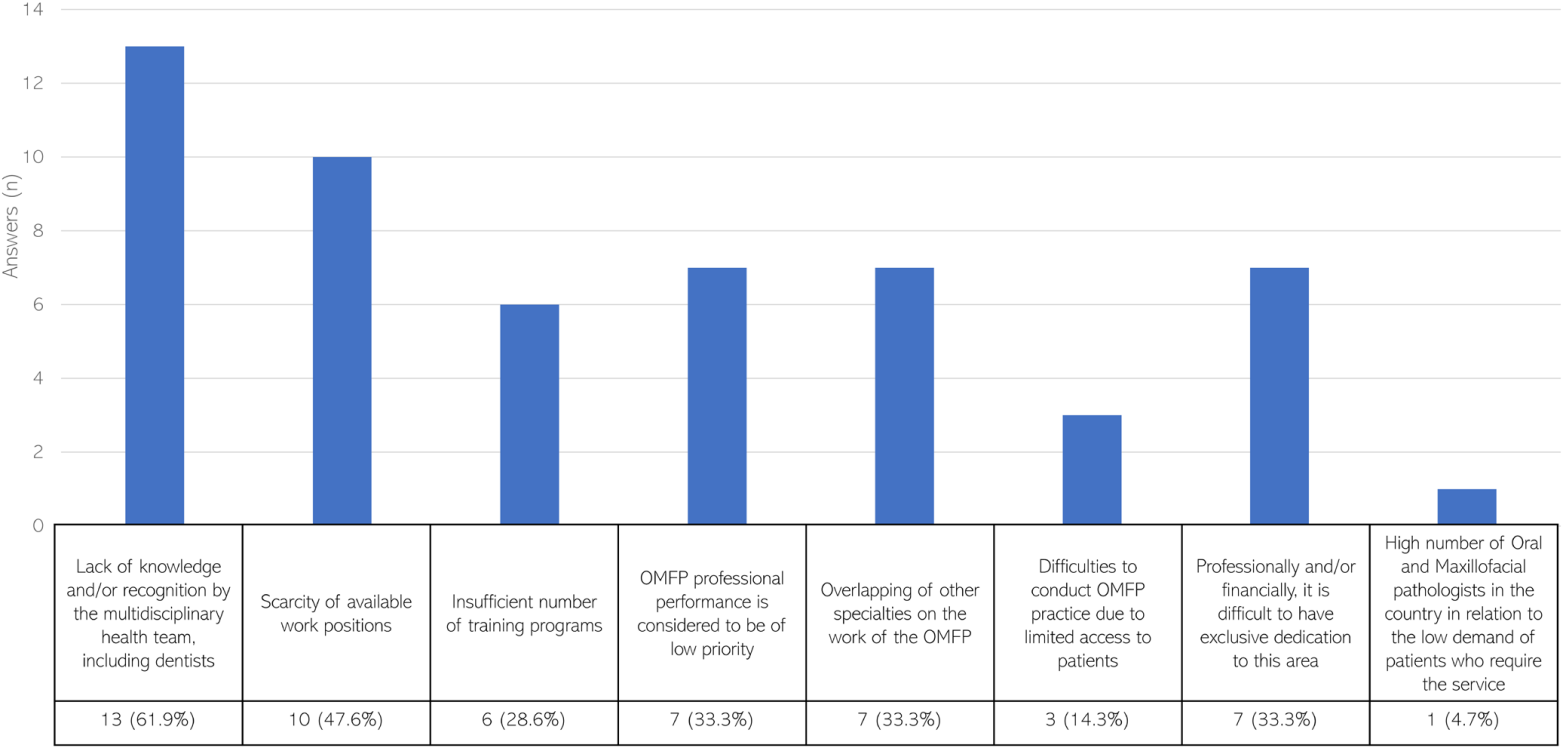
Major barriers to OMFP practice according to the obtained answers.

Experts identified the main individual and general barriers to the OMFP specialty in the countries of the region, with a lack of knowledge or recognition by the multidisciplinary health team representing the most common one (61.9%) (Figure 3).

## 4 Discussion

OMFP has historically developed as a dental specialty related to other medical and dental specialties. However, its scope may be limited or unfamiliar to multidisciplinary teams seeking to optimize patient care [5, 15]. In a previous international study, experts from Brazil and Mexico provided information on demographic aspects, specialty status, and access requirements for training [6]. A more recent study analyzed training and professional practice in 11 Latin American countries [16]. Despite the facts described above, the scope of this study is noteworthy: our research involved OMFP experts from 21 countries and broadly evaluates the OMFP scope considering sociodemographic aspects, training, practice, specialty recognition, and perceived barriers for practice, all key aspects for the exchange of knowledge and potential OMFP development in LAC.

The use of direct and indirect recruitment strategies resulted in a participation rate of 63.6%, comparable to the 66.7% achieved by Rogers et al. [17] and higher than the 52% reported by Hunter et al. [6]. Nonetheless, our data collection tool—in Spanish and English—was self‐managed, and inconsistencies were verified by two researchers (L.V.G.‐P. and C.S.‐S.). This approach ensured 100% completion of the survey by the respondent participants and makes applicable the concept of the “power of information” used in qualitative research. No contact was achieved in some of the Caribbean countries, where participation might have been affected by geographic location, linguistic and cultural differences, and professional interactions, as explained by authors who highlight the ethical, social, and cultural challenges as a hindering factor to obtain a representative sample [18]. The quality of the information was prioritized over the size of the sample, aiming to produce new knowledge [19].

### 4.1 Demographics and Professional Profile of Respondents

The mean age among the 23 participants was 48.8 years (range 31–76 years), unlike the findings reported by Vincent et al., where the average age was 52.3 years with a range of 27–87 years [20]. As for the professional profile, all the participants had postgraduate training, most with specialization degrees and, to a lesser extent, master's and doctoral degrees, 82.6% of which were obtained in an international institution. Although this data is not comparable with similar studies, it is relevant for scientific mobility assessment. It brings advantages related to science development, scientific knowledge dissemination, informed decision making in science management, and training of qualified personnel, and favors the advancement of academic institutions, the increase of their scientific productivity, and the professional success of researchers. Yet, the impact of political, economic, and social factors for each country is unclear [21].

### 4.2 Characterization of the Oral and Maxillofacial Pathology Specialty: Recognition, Quality Standards and Professional Associations

OMFP is recognized as a specialty by a national authority in 76.2% of LAC countries, which is lower than the 90.9% recognition previously reported in LAC [16], but higher than findings from a previous international study [6]. However, the review of quality policies in the region was not assessed, and according to a PAHO report, only 42.9% of the countries in the region had guidelines for the practice of health specialties, while other countries lacked information from the authorities to monitor these policies, making it difficult to make effective decisions related to oral healthcare [10]. Consequently, there is a noteworthy opportunity for cooperation among scientific societies to create protocols that aim to guide management of the OMFP specialty. Such protocols could ensure quality control, increase productivity, reduce turnaround times, provide more accurate diagnostic results, and ultimately improve patient care in LAC countries [2, 3].

### 4.3 Academic Training Programs in Oral and Maxillofacial Pathology Within the Region

One‐third of LAC countries were reported to offer OMFP postgraduate programs, some combined with other diagnostic or clinical fields. These findings concur with Rogers et al., who reported that 33.3% of OM programs were integrated with OMFP [17]. Similarly, Hunter et al. found that only 1% of countries engaged students in patient treatment and formal assignments in OM [6]. More recently, Khoury et al. found two other OMFP programs combined with OM [22]. On one hand, considerations raise on the potential overlap between the OMFP and OM specialists' profiles, and the job profiles required for oral health services. On the other hand, recent technological advancements, such as artificial intelligence, bioinformatics, and precision medicine already being implemented in countries like Brazil, offer an opportunity to define new roles for OMFP professionals in interdisciplinary patient management teams, create clinical guidelines, and promote research initiatives. There is also a valuable opportunity in LAC with its prominent scientific leaders to develop academic training models that foster a clear professional identity for specialists, while growing and diversifying postgraduate programs, supporting globalization, scientific and technological development [22].

### 4.4 Number of Specialists and Professional Practice in LAC Countries

There is a significant difference in the number and distribution of oral and maxillofacial pathologists across the region. However, this information should be treated with caution because it was not obtained from official sources, as evidenced by the discrepancies in some numbers in our study and that of Santos‐Leite et al., conducted in the same region of LAC [16].

The most common practice settings for oral and maxillofacial pathologists in LAC countries were universities (90.5%), private practice, and research environments. Vincent et al. reported that 55% of AAOMP members held positions in dental schools, 14% in medical schools or hospitals, and 17% in private practices [20]. They also noted that 59% of oral surgical pathology laboratories were in dental schools, 18% were in medical schools or hospitals, and 15% were private [20]. On the contrary, Wright et al. found that most oral and maxillofacial pathologists worked in dental schools: 18 respondents worked full‐time and 3 worked part‐time; 5 practitioners worked full‐time in a university hospital, 3 worked in private laboratories, and 3 worked in research. In that study, most respondents did not engage in general dentistry, and the rest saw between 10 and 30 patients monthly [23].

### 4.5 Barriers to Professional Practice

According to the participants, this study identified the lack of recognition by healthcare teams as a key barrier, which may be partly due to the scarcity of OMFP services. Medical pathologists and both general and specialist dentists are often unfamiliar with the OMFP specialty, leading to a lack of referrals or second opinions in complex cases [24, 25]. Regarding job availability and the limited prioritization of OMFP specialists within healthcare services, the literature highlights two main scenarios that hinder the recruitment of qualified professionals: hiring constraints and low salaries in laboratories, and the predominant incorporation into academia. The latter can result in prolonged job stability for a select few while restricting employment opportunities for new faculty members during generational transitions [23, 25, 26]. In terms of overlap with other specialties, Alrashdan et al. indicate that a clear delimitation of OMFP scope of practice is still challenging [27].

Finally, the limited number of OMFP training programs should be interpreted cautiously, given the disparities in professional education and the demand for highly qualified personnel in healthcare systems. This issue has been highlighted by PAHO in its report on healthcare quality strategies for 2020–2025 in LAC [10].

## 5 Limitations

Some limitations from this study must be noted, such as the absence of expert contact information from some of the 33 LAC countries. We acknowledge that the purposive sampling with one representative per country can restrict the generalizability of the results and prevent statistical analysis. Additionally, potential biases inherent to the use of self‐administered surveys can be influential, and the absent assessment of other relevant factors regarding LAC OMFP scope, such as policies, quality, and coverage, highlights the need for further research. Notwithstanding these limitations, considering the limited research in this area, these initial findings serve as a crucial foundation for future studies with a larger sample and a validated instrument.

## 6 Conclusion

This study overviews the training, practice, and recognition of OMFP in 21 LAC countries. The data highlight significant heterogeneity both within the region and against global studies. However, these findings provide a foundation for future research that involves more professionals and countries as well as the evaluation of OMFP competencies, exploring the potential restructuring of postgraduate programs and the potential interest of dental students and graduates in pursuing this specialty.

## Author Contributions

Conceptualization: A.R.S.‐S., L.V.G.‐P., C.S.‐S., A.C.P.R.S., A.L.D.A., K.P.D.G., K.D.H., L.P.A.A. Data curation: L.V.G.‐P., C.S.‐S. Formal analysis: L.V.G.‐P., C.S.‐S. Investigation: L.V.G.‐P., C.S.‐S., A.M.‐T., A.V.O.P., A.B.R.S., C.A.G.N., C.A.T., C.P.P.‐V., E.A.C.G., E.M.C., F.J.M.C.Z., G.G.H., H.R., I.R.H.C., J.L.F.R., K.P.D.G., L.E.K., M.C.G.G., M.L.P., M.V.‐D., P.A.V., P.R.O., R.G.‐M., R.B.‐M., W.A.G.‐A., W.D.A. Methodology: A.R.S.‐S., L.V.G.‐P., C.S.‐S., A.C.P.R.S., A.L.D.A., K.P.D.G., K.D.H., L.P.A.A. Supervision: A.R.S.‐S. Writing – original draft: L.V.G.‐P., C.S.‐S., A.R.S.‐S. Writing – review and editing: L.V.G.‐P., C.S.‐S., A.M.‐T., A.V.O.P., A.B.R.S., C.A.G.N., C.A.T., C.P.P.‐V., E.A.C.G., E.M.C., F.J.M.C.Z., G.G.H., H.R., I.R.H.C, J.L.F.R., K.P.D.G., L.E.K., M.C.G.G., M.L.P., M.V.‐D., P.A.V., P.R.O., R.G.‐M., R.B.‐M., W.A.G.‐A., W.D.A., A.R.S.‐S.

## Conflicts of Interest

The authors declare no conflicts of interest.

## Supporting information


**Data S1:** Supporting Information.

## Data Availability

The data that support the findings of this study are available from the corresponding author upon reasonable request.
