# Supplementary material for: Oral and Maxillofacial Pathology in Latin America and the Caribbean: A Comprehensive Survey of Recognition, Training, and Practice
Source: J Oral Pathol Med. 2025 Sep 3;54(10):1001–11. doi: 10.1111/jop.70051 (PMC12602125; doi:10.1111/jop.70051)
Supplement: Supplementary file 1 — Data S1: Supporting Information. [file JOP-54-1001-s001.docx]

**SUPPLEMENTARY MATERIAL**

**Supplementary material 1.** Latin American and The Caribbean countries according to the Pan American Health Organization, W.H.O (2023). PAHO Countries and Centers. <https://www.paho.org/en/countries-and-Centers>.

| **Latin American and Caribbean countries (n=33)** |
| --- |
| Antigua and Barbuda |
| Argentina |
| Bahamas |
| Barbados |
| Belize |
| Bolivia |
| Brazil |
| Chile |
| Colombia |
| Costa Rica |
| Cuba |
| Dominica |
| Ecuador |
| El Salvador |
| Guatemala |
| Grenada |
| Guyana |
| Haiti |
| Honduras |
| Jamaica |
| Mexico |
| Nicaragua |
| Panama |
| Paraguay |
| Peru |
| Dominican Republic |
| Saint Kitts & Nevis |
| Saint Lucia |
| Saint Vincent & Grenadines |
| Suriname |
| Trinidad & Tobago |
| Uruguay |
| Venezuela |

**Supplementary material 2.** Latin American and The Caribbean participants per country.

| **Participating countries (n=21)** | **Name of the participating professional** |
| --- | --- |
| Argentina | María Luisa Paparella DDS, PhD |
| Belize | Loyden Evan Ken MD |
| Bolivia | Janeth Liliam Flores Ramos DDS |
| Brasil | Pablo Agustin Vargas DDS, PhD |
| Chile** | Ana Verónica Ortega Pinto DDS, MSc  Wilfredo Alejandro González Arriagada DDS, PhD |
| Colombia** | Claudia Patricia Peña Vega DDS, MSc  Carlos Alberto Gaidos Nates DDS |
| Costa Rica | Roberto Gerber Mora DDS, MSc |
| Cuba | Gilda Lucia García Heredia DDS, MSc |
| Ecuador | Patricia Reiván Ortiz DDS, MSc |
| El Salvador | Florence Juana María Cuadra Zelaya DDS, PhD |
| Honduras | Claudette Arambu Turcios DDS |
| Guatemala | Ileana del Rosario Hurtado Castillo DDS, MSc |
| Jamaica | Arvind Babu Rajendra Santosh BDS, MDS |
| Mexico | Adalberto Mosqueda Taylor DDS, MSc |
| Nicaragua | Erick Antonio Castillo Gurdian DDS, MSc |
| Panama | Erick Martínez Cruz DDS |
| Paraguay | María del Carmen González Galván DDS, PhD |
| Peru | Wilson Delgado Azañero DDS, PhD |
| Dominican Republic | Helen Rivera DDS, MSc |
| Uruguay | Ronell Bologna Molina DDS, PhD |
| Venezuela | Mariana Villaroel Dorrego DDS, PhD |
| **No participants (n=12)** |  |
| Antigua and Barbuda |  |
| Bahamas |  |
| Barbados |  |
| Dominica |  |
| Grenada |  |
| Guyana |  |
| Haiti |  |
| Saint Kitts & Nevis |  |
| Saint Lucia |  |
| Saint Vincent & Grenadines |  |
| Suriname |  |
| Trinidad and Tobago |  |

**: In cases with more than one respondent, answers were merged to achieve a consensus per country.

**Supplementary material 3.** Participant’s demographic information and academic training

| **Characteristics** | **N (%)** | |
| --- | --- | --- |
| **Total** | **23 (100)** | |
|  |  | |
| **Gender** |  | |
| Female | 13 | (56.5) |
| Male | 10 | (43.5) |
|  |  | |
| **Age (years)** |  | |
| Mean | 48.8 | |
| Range | 31-76 | |
|  |  | |
| **Location** |  | |
| Current workplace at origin country | 20 | (87.0) |
| Current workplace at different country | 3 | (13.0) |
|  |  |  |
| **Academic background*** |  |  |
| *Specialization* | 18 | (78.3) |
| Oral and Maxillofacial Pathology | 11 | (47.8) |
| Anatomical Pathology | 1 | (4.3) |
| Oral Medicine | 4 | (17.4) |
| Other (smoking cessation and endodontics) | 2 | (8.7) |
|  |  |  |
| *Master’s degree* | 11 | (47.8) |
| Oral and Maxillofacial Pathology | 6 | (26.1) |
| Oral Medicine | 4 | (17.4) |
| Other (Geriatric dentistry) | 1 | (4.3) |
|  |  |  |
| *Doctorate’s degree* | 8 | (34.8) |
| Oral and Maxillofacial Pathology | 6 | (26.1) |
| Oral Medicine | 1 | (4.3) |
| Other (Biomedical sciences) | 1 | (4.3) |
|  |  |  |
| **International academic training** |  |  |
| Yes | 19 | (82.6) |
| No | 4 | (17.4) |
|  |  |  |
| **OMFP training countries*** |  |  |
| Mexico | 4 | (17.4) |
| Brazil | 3 | (13.0) |
| United States | 3 | (13.0) |
| Chile | 3 | (13.0) |
| Colombia | 2 | (8.7) |
| España | 2 | (8.7) |
| United Kingdom | 2 | (8.7) |
| Cuba | 1 | (4.3) |
| Italia | 1 | (4.3) |
|  |  |  |
| **Complementary interdisciplinary training** |  |  |
| Yes | 18 | (78.3) |
| No | 5 | (21.7) |
|  |  |  |
| **Complementary training areas*** |  |  |
| Molecular Biology/Molecular Techniques/Molecular Pathology | 6 | (26.1) |
| Immunohistochemistry | 5 | (21.7) |
| General/human pathology | 3 | (13.0) |
| Surgical/oncological pathology | 3 | (13.0) |
| Histological techniques | 2 | (8.7) |
| Autopsy/forensic pathology | 2 | (8.7) |
| Oral and maxillofacial surgery | 2 | (9.5) |
| Gastroenterology Pathology | 1 | (4.3) |
| Scanning electron microscopy | 1 | (4.3) |
| Cell culture | 1 | (4.3) |
| Cytopathology/Cytology/FNA | 1 | (4.3) |
| Dermatopathology | 1 | (4.3) |
| Orthopedic pathology and soft parts | 1 | (4.3) |

*: Participants were able to select more than one answer.

**Supplementary material 4.** Participant’s professional practice

| **Characteristics** | **N** | **(%)** | |
| --- | --- | --- | --- |
| **Total** | **23** | **(100)** | |
| Professional positions and performance* |  | | |
| Universities (public and private) | 19 | | (82.6) |
| Hospitals (public and private) | 2 | | (8.7) |
| Laboratory practice (public and private) | 22 | | (95.7) |
| Other (member of the Ministry of Health) | 1 | | (4.3) |
|  |  | | |
| What is the general scope of work of the histopathology service you are linked to? * |  | | |
| Oral and maxillofacial biopsies | 21 | | (91.3) |
| Oral and maxillofacial resections | 12 | | (52.2) |
| Biopsies of head and neck sites other than oral cavity | 3 | | (13.0) |
| Resections of head and neck sites other than oral cavity | 3 | | (13.0) |
| Immunohistochemistry | 10 | | (43.5) |
| Genetic / molecular tests | 1 | | (4.3) |
|  |  | | |
| How many samples are received at the histopathology service you are linked to per year? |  |  | |
| < 500 | 13 | (56.5) | |
| 501-1000 | 4 | (17.2) | |
| 1001- 2500 | 2 | (8.7) | |
| 2501- 5000 | 2 | (8.7) | |
| 5001- 10000 | 0 | (0.0) | |
| 10001 or more | 1 | (4.3) | |
| Does not apply | 1 | (4.3) | |
|  |  |  | |
| What is the estimated number of histopathological reports you generate monthly? (Individual metric) |  |  | |
| <50 | 13 | (56.5) | |
| 51-150 | 4 | (17.4) | |
| 151-250 | 4 | (17.4) | |
| 251-350 | 1 | (4.3) | |
| >351 | 0 | (0.0) | |
| Does not apply | 1 | (4.3) | |
|  |  |  | |
| Where does the remuneration for the histopathological work you do comes from? * |  |  | |
| Public financing / Government agencies | 9 | (39.1) | |
| Private financing / Consortia / Insurance | 14 | (60.9) | |
| Specific financing agencies | 0 | (0.0) | |

*: Participants were able to select more than one answer.

**Supplementary material 5.** Name, acronym, year of creation, journals and social networks of the Associations/Federations/Societies

| **COUNTRY** | **NAME** | **ACRONYM** | **YEAR OF CREATION** | **JOURNAL** | **CONTACT / SOCIAL MEDIA** |
| --- | --- | --- | --- | --- | --- |
| Brazil | Brazilian Society of Stomatology and Oral and Maxillofacial Pathology | SOBEP | 1974 | JORDI | estomatologia@sobep.com.br  "@sobep" |
| Colombia | Colombian Academy of Oral Pathology | ACPO | 2003 |  | patologiaoralcolombia@gmail.com  patologos_orales_acpo |
| Chile | Chilean Society of Bucomaxillofacial Pathology | SPBMFCH | 1994 |  | contacto@patologiaoraldhile.cl  patologia.oral.de.chile |
| Mexico | Mexican Association of Pathology and Oral Medicine | AMPMB | 2011 |  | ampmbcolegio@gmail.com |
| Nicaragua | International Oral Medicine Society | SMOI | 2020 | Epithelium | sociedademedicinaoral@gmail.com |
| Paraguay | Paraguayan Society of Pathology and Oral Medicine | SPPMB | 2016 |  | sppmb@secretaria@gmail.com |
| Peru | Peruvian Association of Pathology and Oral and Maxillofacial Medicine | APPSMED | 2007 |  | wilson.delgado@upch.pe |
| Uruguay | Uruguayan Society of Stomatological Pathology | SUPE | 2010 |  | svcelhay@adinet.com.uy |
| Venezuela | Venezuelan Society of Oral Medicine | SVPB | 2005 |  | @svmedicinabucal |

**Supplementary material 6.** Areas of combination in LAC’s OMFP postgraduate training.

| **Areas*** | **N (%)** |
| --- | --- |
|  | **6 (100)** |
| Oral Medicine (Brazil, Chile, Mexico and Peru) | 4 (66.0) |
| Imaging and Radiology (Colombia) | 1 (16.7) |
| Oral Surgery (Colombia) | 1 (16.7) |

*: Participants were able to select more than one answer.
